# Supplementary material for: Overhauser dynamic nuclear polarization (ODNP)-enhanced two-dimensional proton NMR spectroscopy at low magnetic fields
Source: Magn Reson (Gott). 2021 Apr 12;2(1):117–28. doi: 10.5194/mr-2-117-2021 (PMC9030190; doi:10.5194/mr-2-117-2021)
Supplement: The supplement related to this article is available online at: https://doi.org/10.5194/mr-2-117-2021-supplement. [file mr-2-117-supplement.pdf]

Supplement of Magn. Reson., 2, 117–128, 2021
https://doi.org/10.5194/mr-2-117-2021-supplement
© Author(s) 2021. CC BY 4.0 License.

Open Access
MAGNETIC
RESONANCE

Supplement of

Overhauser dynamic nuclear polarization (ODNP)-enhanced two-dimensional proton NMR spectroscopy at low magnetic fields

Timothy J. Keller and Thorsten Maly

Correspondence to: Thorsten Maly (tmaly@bridge12.com)

The copyright of individual parts of the supplement might differ from the article licence.

1. ODNP Spectrometer

The ODNP spectrometer used for experiments is shown in **Figure S1**. Key components of the spectrometer are numbered in the figure: (1) Bridge12 microwave power source (MPS) (2) Kea spectrometer (3) RF amplifier (4) shim and B_0 power supplies (5) incubator (6) SABR magnet (7) ODNP resonator (8) remote tuning box. The compact system occupies a 24” x 40” x 32” space. Further reduction in space can be achieved by reducing the size of the shim power supplies.

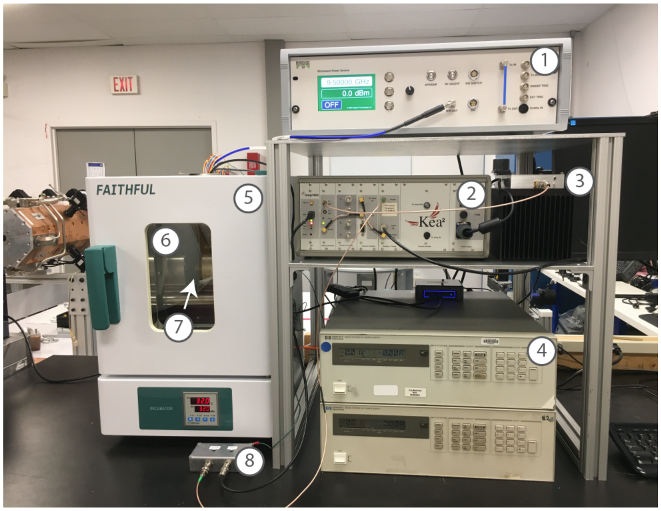

Figure S1: ODNP Spectrometer used for measurements. Refer to text for numbered components.

2. Linewidth Measurements

The NMR spectrum of water with 200 μM TEMPOL sample is shown in Figure S2. The water peak was referenced to 4.8 ppm. The water spectrum has a full width half max (FWHM) linewidth of 2.3 Hz. (0.16 ppm). The enhancement at 33 dBm microwave power was determined to be -19. The broad baseline features indicate higher order inhomogeneities, which would require additional higher order shims to correct. Lower concentrations of polarizing agent produce much narrower lines but result in lower enhancements.

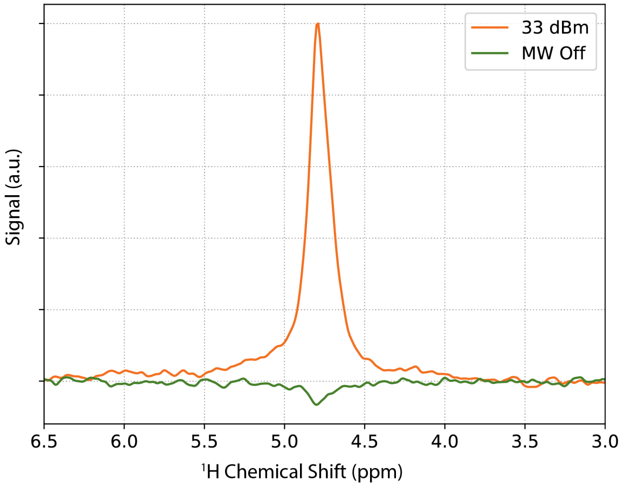

Figure S2: Single shot NMR spectrum of water with 200 µM TEMPOL. A line broadening of 1 Hz was applied and data was zero filled to 4 times original length.

The linewidth for the ethyl crotonate spectra were obtained by fitting the methyl protons to the sum of four Voigt distributions. The Lorentzian linewidth (γ) and the Gaussian linewidth (σ) for all peaks were assumed to be the same. The amplitude and position of each peak were free parameters. The experimental data and fits are shown in **Figure S3**.

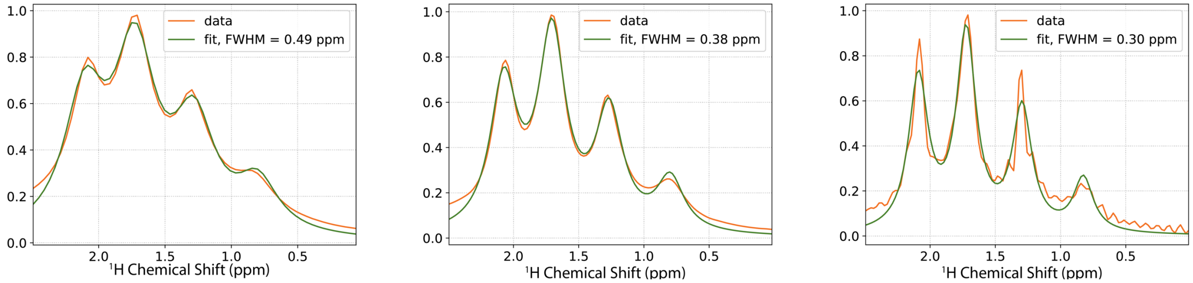

Figure S3: Ethyl crotonate spectra used for linewidth measurements. The experimental data is shown in orange and the fit is shown in green. The calculated FWHM linewidth in ppm for each fit is indicated in the legend of the figure. ODNP enhanced ethyl crotonate NMR spectra with 10 mM TEMPONE using apodization function: (left) 1 Hz Lorentzian line broadening (middle) Gauss-Lorentz apodization with $\boldsymbol{\sigma}$ = 4 Hz and $\boldsymbol{\gamma}$ = 4 Hz (right) resolution enhancement with $\boldsymbol{T_2^*}$ of 0.2 s.

Although the true number of methyl peaks is 5, the two overlapping peaks at 1.7 ppm are close in chemical shift value and are fit with a single Voigt distribution. The JRES data were processed using a Lorentz-Gauss transformation because it removes the long tail of the Lorentzian distribution.

3. Ethyl Crotonate Phase Correction:

In addition to compensating for any magnetic field drift, a phase correction was also applied to the 2D JRES spectrum. The phase was determined by applying a zeroth order phase correction to the interleaved reference spectra. The phase for each JRES slice was then corrected with the corresponding phase for the 1D spectra. The phase varies approximately linearly over the course of the experiment as shown in **Figure S4**. If not corrected, the phase drift would result in a frequency offset in the indirect dimension. We attribute the phase drift to acquisition delay which will result in a linear phase roll in frequency domain as the field drifts. Additionally, instrumental and environmental instabilities (e.g. temperature fluctuations in the room) can cause long term phase drift. All these effects can be corrected for by interleaved spectral referencing.

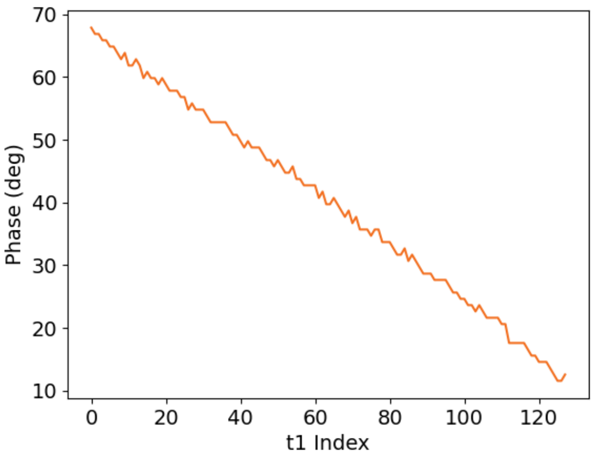

Figure S4: Phase drift over the ethyl crotonate JRES experiment.

4. ODNP Enhanced JRES of Ethanol

As the second example we performed ODNP-enhanced JRES spectroscopy using a sample of neat ethanol with 10 mM TEMPOL. Analogous to the ethyl crotonate JRES spectrum, the ethanol JRES spectrum was corrected for field drift and phase drift. The interleaved 1D proton spectra used for the ethanol field drift correction are shown in **Figure S5 (a)**. The field drift was typical of a 30 minute experiment at about 60 ppm. The 4th degree polynomial fit to the field drift is shown in **Figure S5 (b)** and the residual of the fit is shown in **Figure S5 (c)**.

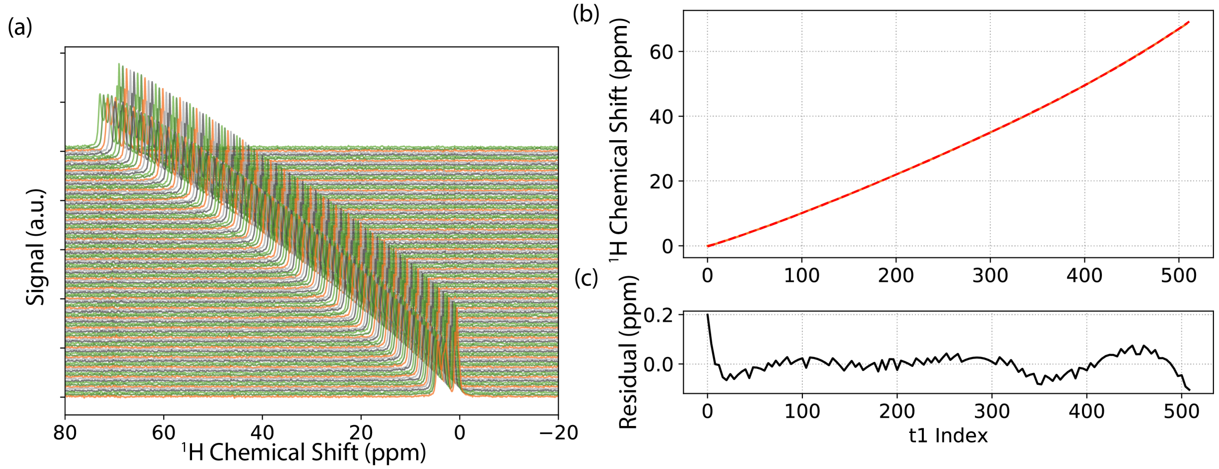

Figure S5: (a) 1D ethanol spectra used for field drift correction. (b) Calculated chemical shift and 4th degree polynomial fit. (c) Residual of 4th degree polynomial fit.

The JRES spectrum for ethanol with 10 mM TEMPOL is shown in **Figure S6 (a)**. The slices showing the resonances for individual sites are shown in **Figure S6 (b).** As expected, we observe a triplet for the protons of the methyl group (A protons) at a chemical shift of 1.2 ppm and a quartet for the methylene protons at 3.5 ppm (B protons). A broad resonance is observed at a chemical shift of 5.1 ppm, which is assigned to the proton of the hydroxyl group (C protons). This peak is significantly broadened due to the exchange rate of the OH protons. In the absence of exchange, this peak would be much narrower. In addition, the quartet observed at 5.1 ppm would be observed as a doublet of quartet. However, due to the proton exchange this multiplet is observed as a broadened quartet.

Features of strong coupling between the methyl and methylene groups can be clearly distinguished in the 2D JRES spectrum and are located at a chemical shift of 2-3 ppm and around a J-coupling of ±15 Hz (see **Figure S6 (a)**, dashed box). The broad features at 4-5 ppm and a J-coupling of ±10 Hz can be attributed to strong coupling peaks of the methylene protons and the proton of the hydroxy group. Individual slices for the different proton sites and the resulting coupling patterns are shown in **Figure S6 (b)**. Overall, all expected features can be observed in these slices.

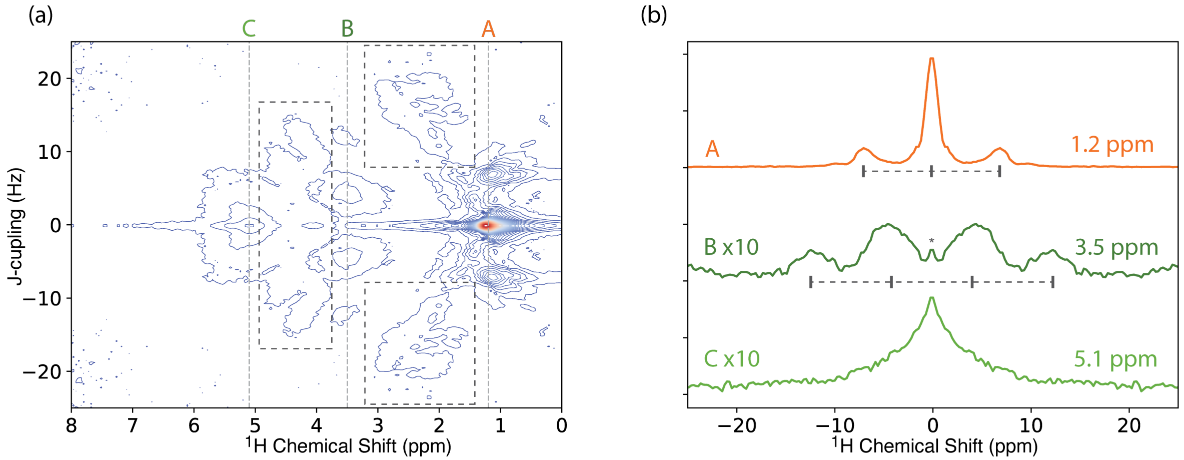

Figure S6: (a) JRES spectrum of neat ethanol with 10 mM TEMPOL. Signals shown in the dashed box are attributed to strong coupling effects. (b) JRES slices for ethanol from (a). Features marked with an asterisk are artifacts of the JRES experiment.

The skyline projection and 1D proton spectrum for ethanol with 10 mM TEMPOL are shown in **Figure S7**. The 1D proton spectra are broadened by the viscosity of the solution and polarizing agent, however, it is still possible to resolve the J-couplings in the 1D proton spectrum. The CH_3 triplet is visible at 1.2 ppm. Similarly to the JRES slice, the CH_2 group is a broadened quartet at 3.5 ppm. The OH peak is visible as a broad peak at 5.1 ppm. The skyline projection is not quantitative. The CH_2 and OH peaks are visible, but significantly reduced in amplitude. A quantitative analysis could be obtained by integration of the peaks. Integration would allow for accurate measurement of enhancements for each chemical site.

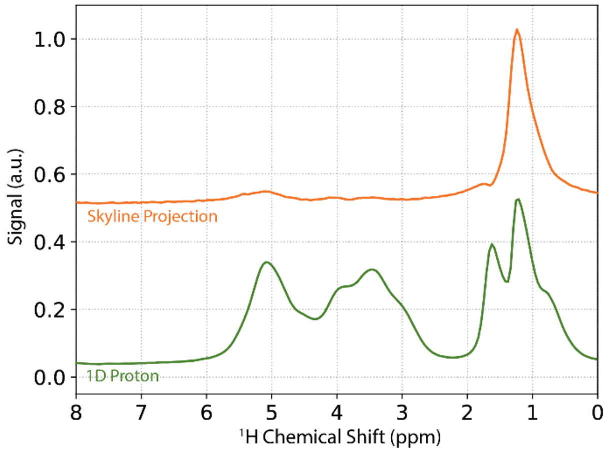

Figure S7: NMR spectra of ethanol with 10 mM TEMPOL (top) JRES skyline projection (bottom) 1D proton spectrum. Spectra are offset for clarity.

5. Additional Window Functions

To process the JRES data, a Lorentz-Gauss transformation with equal Lorentzian and Gaussian linewidths was used. Such an apodization function will have minimal effect on the linewidth, but removes the long tail from the Lorentzian distribution.

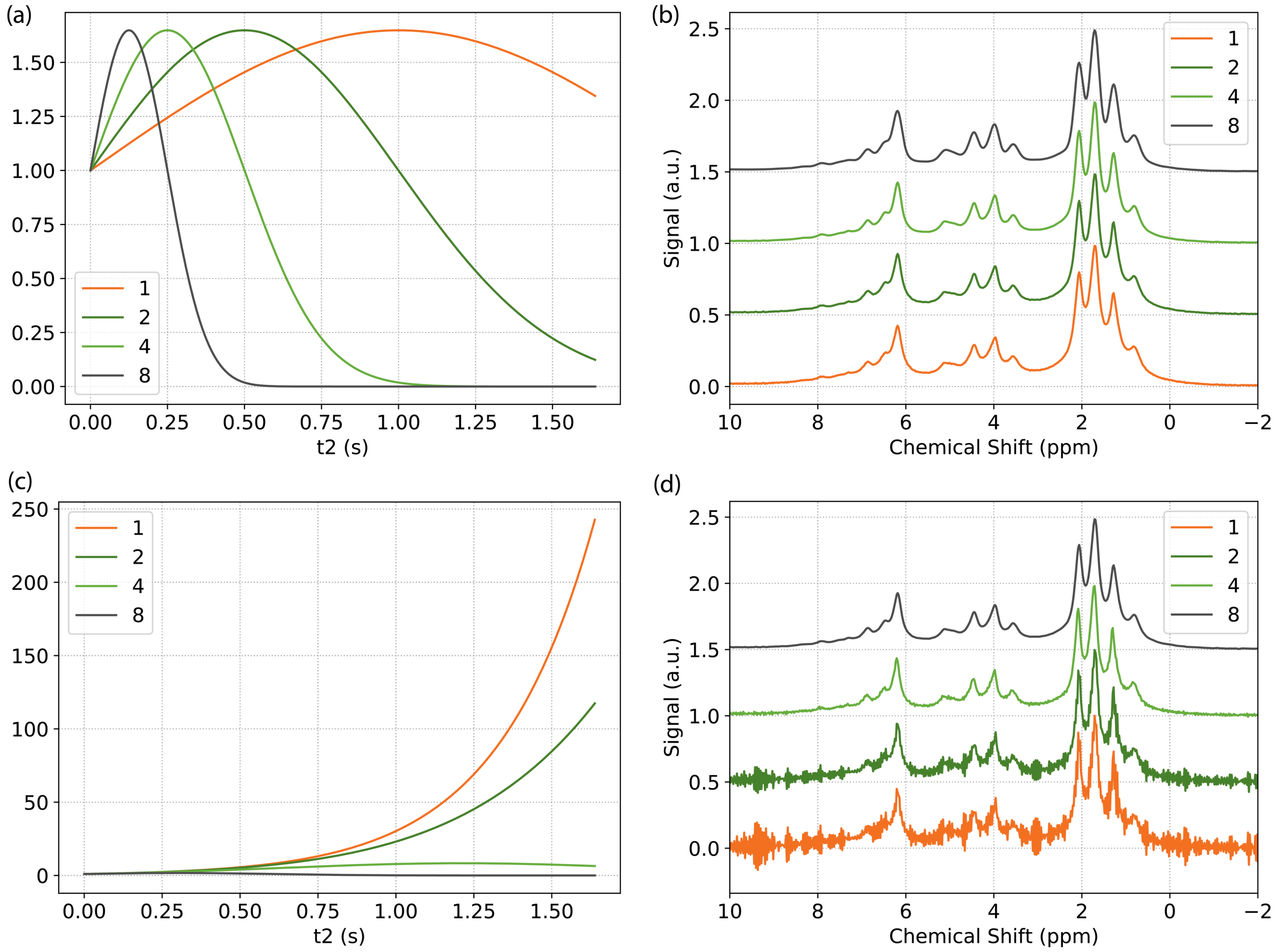

Figure S8: (a) Lorentz-Gauss apodization functions using equal Lorentzian and Gaussian linewidths and (b) corresponding ethyl crotonate spectra. (c) Lorentz-Gauss apodization functions using matched Lorentzian filter (7 Hz) and variable Gaussian linewidths. The linewidth in Hz for each trace is indicated in the legend of each figure.

In **Figure S8**, we show the ethyl crotonate spectra using a Lorentz-Gauss transformation with equal Lorentzian and Gaussian linewidth parameters. The apodization function for each spectrum is shown in **Figure S8 (a)**. The corresponding spectrum is shown in **Figure S8 (b)**. As can be seen from the spectra, there is minimal change in linewidth, however, the lines will begin to broaden as larger apodization linewidths are used. In **Figure S8 (c-d)**, we use a matched filter for the Lorentzian linewidth of 7 Hz and vary the Gaussian linewidth. In this case, improved spectral resolution can be achieved, but only at the expense of signal to noise.

In addition, we show the effect of using a Hamming window for apodization in **Figure S9**. The ethyl crotonate linewidth obtained by using a Hamming window was 6.7 Hz (0.45 ppm).

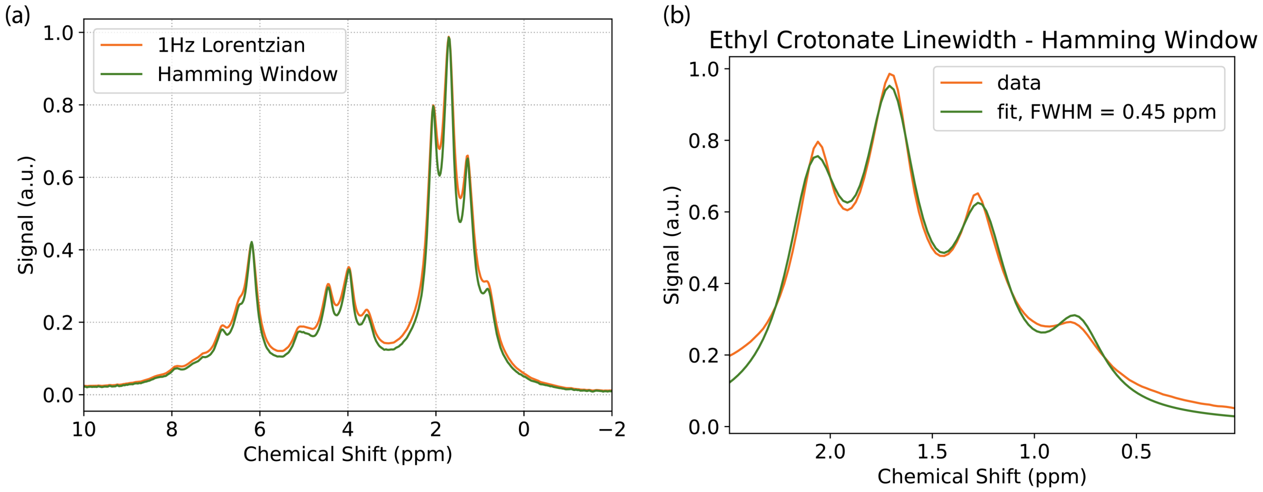

Figure S9: (a) Comparison of ethyl crotonate with 10 mM TEMPONE linewidth using a 1 Hz Lorentzian apodization function (orange) and Hamming window (green). (b) Fit of methyl peaks for ethyl crotonate spectrum with Hamming window applied using the sum of 4 Voigt distributions.
